# Laminarin Attenuates Ultraviolet-Induced Skin Damage by Reducing Superoxide Anion Levels and Increasing Endogenous Antioxidants in the Dorsal Skin of Mice

**DOI:** 10.3390/md18070345

**Published:** 2020-06-30

**Authors:** Ji Hyeon Ahn, Dae Won Kim, Cheol Woo Park, Bora Kim, Hyejin Sim, Hyun Sook Kim, Tae-Kyeong Lee, Jae-Chul Lee, Go Eun Yang, Young Her, Joon Ha Park, Tae Heung Sim, Hyun Sam Lee, Moo-Ho Won

**Affiliations:** 1 Department of Biomedical Science, Research Institute of Bioscience and Biotechnology, Hallym University, Chuncheon, Gangwon 24252, Korea; jh-ahn@hallym.ac.kr (J.H.A.); xorud312@naver.com (T.-K.L.); 2 Department of Neurobiology, School of Medicine, Kangwon National University, Chuncheon, Gangwon 24341, Korea; flfhflfh@naver.com (C.W.P.); nbrkim17@gmail.com (B.K.); janny20@naver.com (H.S.); anajclee@kangwon.ac.kr (J.-C.L.); 3 Department of Biochemistry and Molecular Biology, and Research Institute of Oral Sciences, College of Dentistry, Gangnung-Wonju National University, Gangneung, Gangwon 25457, Korea; kimdw@gwnu.ac.kr; 4 Leefarm Co., Ltd., Hongcheon, Gangwon 25117, Korea; K18860@naver.com (H.S.K.); 119ato@naver.com (T.H.S.); 5 Department of Radiology, Kangwon National University Hospital, Chuncheon, Gangwon 24289, Korea; yangke@kangwon.ac.kr; 6 Department of Dermatology, Kangwon National University Hospital, Kangwon National University School of Medicine, Chuncheon, Gangwon 24289, Korea; youngderma@knuh.or.kr; 7 Department of Anatomy, College of Korean Medicine, Dongguk University, Gyeongju, Gyeongbuk 38066, Korea; jh-park@dongguk.ac.kr

**Keywords:** antioxidants, collagen fiber, epidermal thickness, laminarin, oxidative stress, UVB

## Abstract

A number of studies have demonstrated that marine carbohydrates display anti-oxidant, anti-melanogenic, and anti-aging activities in the skin. Laminarin (LA), a low-molecular-weight polysaccharide, is found in brown algae. The benefits of LA in ultraviolet B (UVB) induced photodamage of the skin have not been reported. The aim of this study was to investigate the effects of pre-treated LA on histopathological changes and oxidative damage in mouse dorsal skin on day 5, following repeated UVB exposure. Histopathology, Western blot analysis and immunohistochemical studies showed that epidermal thickness in the UVB group was significantly increased; however, the thickness in the UVB group treated with LA (LA/UVB group) was less compared with that of the UVB group. Collagen fibers in the dermis of the UVB group were significantly decreased and destroyed, whereas, in the LA/UVB group, the density of collagen fibers was significantly increased compared with that of the UVB group. Oxidative stress due to superoxide anion production measured via dihydroethidium fluorescence staining was dramatically increased in the UVB group, whereas in the LA/UVB group, the oxidative stress was significantly decreased. Expressions of SOD1, glutathione peroxidase and catalase were markedly reduced in the UVB group, whereas in the LA/UVB group, they were significantly higher along with SOD2 than in the control group. Taken together, our results indicate that LA pretreatment prevents or attenuates skin damage, by decreasing oxidative stress and increasing antioxidant enzymes in mouse dorsal skin.

## 1. Introduction

Skin is made up of two layers, the epidermis and dermis, and serves as an initial barrier against ultraviolet (UV) light, pathogens, and chemicals [1]. In the epidermis, keratinocytes are the major cell type, which play important roles in the skin barrier by secreting various proteins and lipids [2]. The dermis is a fibrous layer composed of collagen and elastin fibers and includes an extracellular matrix composed of molecules such as glycosaminoglycans [3]. Fibroblasts are the major cells in the dermis and mediate the synthesis and degradation of fibrous and non-fibrous matrix proteins [3]. 

Exposure to UV irradiation triggers an acute skin response, including sunburn (erythema), tanning (pigmentation), damage-related immunosuppression and epidermal hyperplasia [4]. Repeated exposure to UV generates excessive reactive oxygen species (ROS) and damages the enzymatic and non-enzymatic antioxidant defense systems in the skin, and results in the damage of cellular proteins, lipids and DNA [5,6]. In addition, UV exacerbates an inflammatory response in the skin by promoting and activating inflammatory cells, such as neutrophils, macrophages, mast cells which cause skin damage (photoaging) [5,6]. 

To prevent UVB-induced skin damage and enhance the integrity of the skin barrier, several studies have reported the development of new bioactive compounds, which are safe and efficacious, from natural sources, instead of synthetic compounds [7,8]. Marine algae are considered an essential source of carbohydrates, proteins, flavonoids and minerals that promote skin health [9]. For example, polysaccharides extracted from marine algae exert antioxidant, anti-inflammatory, anti-atopic and anti-melanogenic effects in in vivo and in vitro studies [9]. 

Among the polysaccharides extracted from marine algae, fucoidan, as a fucose-rich sulfated polysaccharide, is found in the cell wall matrix of several brown algae [10,11]. Studies have reported that fucoidan is effective in strengthening the integrity of skin barrier [12] and in decreasing the symptoms of atopic dermatitis [13] in animal models. Laminarin (LA) is a water-soluble polysaccharide derived from brown algae, such as *Laminaria digitate*, *Ascophyllum nodosum* and *Laminarina hyperborean*, and exhibits anti-oxidant and antibacterial activities [14].

To date, sunblock products have been in great demand, due to effective sun tanning, and for protection against UVB exposure [7]. In this regard, the radioprotective effects of numerous natural resources have been investigated [7,15]. However, a few in vivo studies investigating the benefits of LA against photoaging have reported that LA regulates collagen metabolism in photoaging mouse skin [16]. Therefore, in the present study, we investigated the effects of pre-treatment with LA on histopathological changes, oxidative stress and endogenous antioxidants in mouse dorsal skin, following exposure to repeated UVB irradiation.

## 2. Results

### 2.1. Epidermal Thickness and Dermal Collagen Fibers

**Figure 1:**
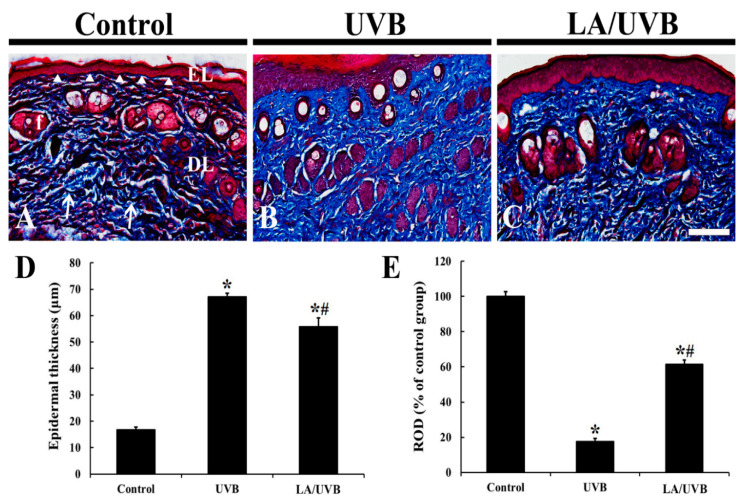
(**A**–**C**) Masson trichrome staining in the control (**A**), UVB (**B**) and Laminarin (LA)/UVB (**C**) groups at 5 days after UVB exposure. The thickness of the epidermal layer (EL, arrow heads) is the thickest in the UVB group, but significantly decreased in the LA/UVB group, as compared to that in the UVB group. Collagen fibers (arrows) stained with dark blue dye are densely distributed in the control group. In the UVB group, collagen fibers are significantly reduced, whereas collagen fibers in the LA/UVB group are significantly increased compared to that in the UVB group. DL, dermal layer; EL, epidermal layer; f, hair follicle. Scale bar = 100 μm. (**D**,**E**) The mean value of epidermal thickness (**D**) and relative optical density (ROD) of collagen fibers in the dermis (**E**) at 5 days after UVB irradiation. The bars indicate the means ± SEM (*n* = 7/group; * *p* < 0.05, vs. control group, # *p* < 0.05 vs. UVB group).

On day 5 after UVB exposure, changes in the thickness of the epidermis and collagen fibers in the dermis in the dorsal skin were evaluated using Masson trichrome staining (Figure 1). The epithermal keratinocytes were stained with red dye in all groups (Figure 1A–D). The epidermal thickness in both UVB and LA/UVB groups was significantly increased (about 3.98 fold and 3.32 fold, respectively) compared to that in the control group; however, the epidermal thickness of the LA/UVB group was less thick (about 83.2%) compared to that of the UVB group (Figure 1A–D).

Collagen fibers in the dermis of the control group were stained with blue dye and abundantly found (Figure 1A), showing that thin and dense collagen bundles were easily observed throughout the dermis (Figure 1A). In the UVB group, dense collagen bundles stained with dark blue dye were remarkably decreased (about 17.7% of the control group) compared to those in the control group, showing that collagen fibers were destructed (Figure 1B,E). However, in the LA/UVB group, the density of collagen fibers was increased (about 61.4% of the control group) compared to that in the UVB group (Figure 1C,E).

### 2.2. Dihydroethidium (DHE) Fluorescence

**Figure 2:**
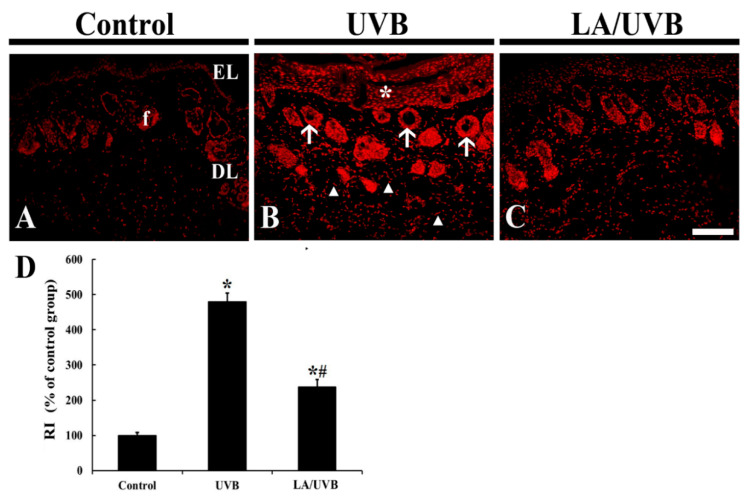
(**A**–**C**) Dihydroethidium (DHE) fluorescence staining in the control (**A**), UVB (**B**) and LA/UVB (**C**) groups at 5 days after UVB exposure. In the UVB group, DHE fluorescence is significantly increased in the epidermis (EL) (asterisk) and dermal hair follicles (f) (arrows) and collagen-like fibers (arrowheads). However, in the LA/UVB group, DHE fluorescence in the structures is significantly lower than that in the UVB group. DL, dermal layer. Scale bar = 100 μm. (**D**) RI of DHE fluorescence structure at 5 days after UVB irradiation. The bars indicate the means ± SEM (*n* = 7/group; * *p* < 0.05 vs. control group, # *p* < 0.05 vs. UVB group).

The change of superoxide anion in the dorsal skin tissue was evaluated by DHE fluorescence staining (Figure 2). In the control group, weak DHE fluorescence was observed mainly in the epidermal layer consisting of keratinocytes and hair follicles, located in the dermis (Figure 2A). In the UVB group, very strong DHE fluorescence was shown in the epidermal keratinocytes, and the dermal hair follicles and fibroblasts at five days after UVB exposure, showing that the relative intensity (RI) of the DHE fluorescence was 479.8% of that in the control group (Figure 2B,D). In the LA/UVB group, the RI of the DHE fluorescence was significantly decreased (49.5% of the UVB group) compared to that in the UVB group (Figure 2C,D). 

### 2.3. Endogenous Antioxidants

#### 2.3.1. SOD1 and SOD2 Protein Levels

**Figure 3:**
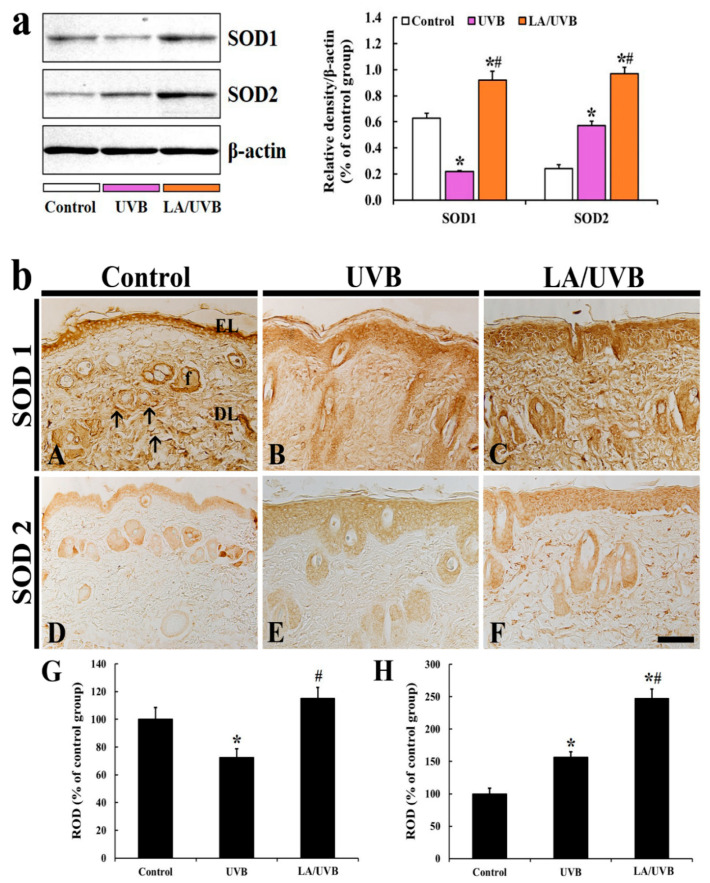
(**a**) Representative blot images and quantitative analysis of SOD1 and SOD2 protein levels in the dorsal skin of the control, UVB and LA/UVB groups at 5 days after UVB irradiation. Bars indicate the means ± SEM (*n* = 7 at each group, * *p* < 0.05 vs. control group, # *p* < 0.05 vs. UVB group). (**b**) SOD1 (**A**–**C**) and SOD2 (**D**–**F**) immunohistochemistry in the dorsal skin in the control (**A**,**D**), UVB (**B**,**E**) and LA/UVB (**C**,**F**) groups at five days after UVB exposure. In the control group, SOD1 immunoreactivity is shown in the epidermal layer (EL), hair follicles (f) and fiber-like structures (arrows). SOD1 immunoreactivity in these structures is decreased in the UVB group, but significantly higher in the LA/UVB group than the control group. SOD2 immunoreactivity in the control group is found in the EL and f. SOD1 immunoreactivity in the structures is increased in the UVB group and more increased in the LA/UVB group than the control group. DL, dermal layer. Scale bar = 100 μm. (**G**,**H**) ROD of SOD1 (**G**) and SOD2 (**H**) immunoreactive structures in all groups at five days after UVB irradiation. The bars indicate the means ± SEM (*n* = 7/group; * *p* < 0.05, vs. control group, # *p* < 0.05 vs. UVB group).

The SOD1 protein level in the dorsal skin of the UVB group was significantly reduced (34.6% of the control group) at 5 days after UVB irradiation, but the SOD1 level in the LA/UVB group was significantly increased (146.9% of the control group) compared to that in the control group (Figure 3a). 

The SOD2 protein level in the dorsal skin of the UVB group was significantly increased (234.3% of the control group) at 5 days after UVB exposure, and, in the LA/UVB group, the SOD2 level was much more increased (398.2% of the control group) in comparison to that in the control group (Figure 3a). 

#### 2.3.2. SOD1 Immunoreactivity 

In the control group, SOD1 immunoreactivity in dorsal skin was predominantly shown in the cytoplasm of the epidermal keratinocytes, hair follicles and fiber-like structures (collagens) in the dermis (Figure 3A). In the UVB group, SOD1 immunoreactivity was significantly decreased (72.6% of the control group) throughout all layers of the dorsal skin compared to that in the control group (Figure 3B,G). However, SOD1 immunoreactivity in the LA/UVB group was higher (115.2% of the control group) than that in the control group (Figure 3C,G).

#### 2.3.3. SOD2 Immunoreactivity 

SOD2 immunoreactivity in the control group was observed in the epidermis and hair follicles in the dermis (Figure 3D). In the UVB group, increased SOD2 expression (156.6% of the control group) was shown in the epidermis and hair follicles (Figure 3E,H). In addition, SOD2 immunoreactivity in the LA/UVB group was more significantly increased (247.3% of the control group) in the epidermis and dermal hair follicles in comparison to that in the control group (Figure 3F,H). 

#### 2.3.4. Glutathione Peroxidase (GPx) and Catalase (CAT) Protein Levels

**Figure 4:**
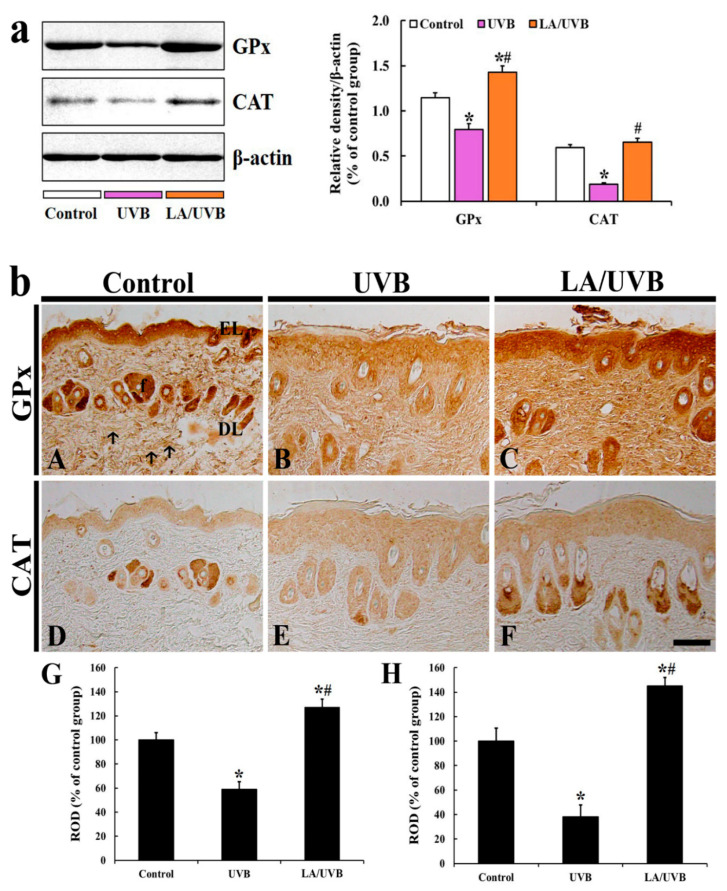
(**a**) Representative blot images and quantitative analysis of GPx and CAT protein levels in the dorsal skin of the control, UVB and LA/UVB group at five days after UVB irradiation. Bars indicate the means ± SEM (*n* = 7 at each group, * *p* < 0.05 vs. control group, # *p* < 0.05 vs. UVB group). (**b**) GPx (**A**–**C**) and CAT (**D**–**F**) immunohistochemistry in the dorsal skin in the control (**A**,**D**), UVB (**B**,**E**) and LA/UVB (**C**,**F**) group at five days after UVB exposure. Strong GPx immunoreactivity in the control group is shown in the epidermal layer (EL), hair follicles (f) and fiber-like structures (arrows). GPx immunoreactivity in the structures of the UVB group is significantly decreased, but significantly higher in the LA/UVB group than the control group. CAT immunoreactivity in the control group is shown in the EL and f. CAT immunoreactivity in the UVB group is significantly decreased but CAT immunoreactivity in the LA/UVB group is significantly higher than that in the control group. DL, dermal layer. Scale bar = 100 μm. (**G**,**H**) ROD of GPx (**G**) and CAT (**H**) immunoreactive structures in all groups at 5 days after UVB irradiation. The bars indicate the means ± SEM (*n* = 7/group; * *p* < 0.05, vs. control group, # *p* < 0.05 vs. UVB group).

GPx protein level in the dorsal skin of the UVB group was significantly decreased (69.7% of the control group) at five days after UVB irradiation, but GPx level in the LA/UVB group was significantly higher (124.9% of the control group) than that in the control group after UVB irradiation (Figure 4a). 

The CAT protein level in the dorsal skin of the UVB group was also significantly decreased (31.9% of the control group) at five days after UVB exposure in comparison to that in the control group, but the CAT level in the LA/UVB group was slightly higher (109.4% of the control group) than that in the control group (Figure 4a). 

#### 2.3.5. GPx Immunoreactivity 

Strong GPx immunoreactivity in the dorsal skin of the control group was shown in the epidermal keratinocytes and dermal hair follicles and fiber-like structures (collagen fibers) (Figure 4A). In the UVB group, GPx immunoreactivity in the structures was significantly decreased (59.1% of the control group) at 5 days after UVB irradiation, compared to that in the control group (Figure 4B,G). However, GPx immunoreactivity in the LA/UVB group was significantly increased (126.9% of the control group) compared to that in the control group (Figure 4C,G). 

#### 2.3.6. CAT Immunoreactivity 

In the control group, weak or intermediate CAT immunoreactivity was detected in the epidermis and the dermal hair follicles (Figure 4D). In the UVB group, CAT immunoreactivity in the epidermis was not changed, but CAT immunoreactivity in the hair follicles was significantly decreased (38.3% of the control group) compared to that in the control group (Figure 4E,H). However, in the LA/UVB group, CAT immunoreactivity in the hair follicles was significantly increased (145.1% of the control group) compared to that in the control group, showing that CAT immunoreactivity in the epidermis was also similar to that in the control group (Figure 4F,H).

## 3. Discussion

Exposure to UV radiation is the primary factor leading to the skin aging that is known as photoaging [17]. Solar UVB radiation with a wavelength range of 290–320 nm penetrates the epidermis of the skin upon excessive exposure to the sun’s UVB rays [18]. In the present study, we investigated whether LA pretreatment, which has been reported to display antioxidant [19,20] and anti-aging activities [16], attenuates skin damage by reducing oxidative stress and increasing endogenous antioxidant levels in mouse dorsal skin following UVB irradiation. 

Photodamaged skin is generally characterized by an increase in the thickness of the epidermis and loss of mature collagen fibers in the dermis [21]. Recently, Deng et al. (2019) reported that the thickness of epidermal layer was dramatically increased with irregular epidermal hyperplasia after UVB irradiation in in vitro and in vivo studies. In the case of collagen fibers in the dermis, type I collagen level [22] or acid-soluble collagen in mice [23] was significantly decreased after treatment with UVB irradiation. Furthermore, the level of matrix metalloproteinase (MMP)-1, an enzyme capable of degrading dermal collagens, was significantly increased after UVB exposure in mice, showing that the level of type I collagen expression was significantly reduced [24]. In our current study, to identify UVB-induced skin damage, a microscopic evaluation of epidermal thickness and collagen fiber expressions was carried out using Masson’s trichrome staining. The results showed that the epidermis in the UVB group was significantly thickened, whereas in the LA/UVB group, the thickened epidermis was significantly attenuated compared with that of the UVB group. In addition, collagen fibers in the UVB group were markedly damaged but attenuated in the LA/UVB group. These results indicate that UVB-induced epidermal hyperplasia and collagen destruction are general features following UVB irradiation, and that the damage is attenuated by LA pretreatment. 

Several studies reported the mechanisms of LA in the skin exposed to UV irradiation. Li et al. (2013) reported that the intraperitoneal injection of LA (5 mg/kg) after UVA and UVB exposure significantly enhanced the level of hydroxyproline, a major component of collagen, together with a decrease in MMP-1 mRNA level in mouse skin [16]. In addition, Ayoub et al. (2015) demonstrated that LA extracted from brown seaweed *Saccharina longicruris* increased collagen I levels in the dermis generated via tissue-engineering [25]. LA, a low-molecular-weight polysaccharide (approximately 5 kDa) [26], is a storage β-glucan (glucose-based polysaccharide) [27]. The topical application of β-glucan has been proven to directly increase collagen biosynthesis (type I and III procollagen mRNA and hydroxyproline) by dermal fibroblasts, via nuclear factor-1 (NF-1) activation [28]. Based on the results of previous and current studies, it is likely that the β-glucan component of LA prevents the progression of skin aging, by increasing collagen synthesis in the dermis of UVB-damaged skin.

It has been reported that UVB exposure induces the production of reactive oxygen species (ROS), such as superoxide radical and hydroxyl, and upregulates the intracellular signaling pathways mediated by epidermal growth factor receptor (EGFR) and transcription factors (nuclear factors AP-1 and NF-κB), which activate MMPs, block collagen gene expression and activate keratinocyte proliferation [29]. In this study, we evaluated the production of ROS induced by UVB exposure via DHE histofluorescence staining, which is a useful indicator of the in situ generation of superoxide anion [30]. In our present study, we found a significant increase in superoxide anion production in the skin of the UVB group. However, in the LA/UVB group, the superoxide anion production was dramatically decreased. In addition, in the LA/UVB group, the expression of endogenous antioxidant enzymes (SOD1, SOD2, GPx and CAT) in the dorsal skin was higher than in the UVB group. To the best of our knowledge, this is the first finding reporting the antioxidant efficacies of LA in the skin following UVB irradiation. It has been well known that endogenous antioxidant enzymes, such as SODs, GPx, and CAT, scavenge ROS [31]. Recently, Xian et al. (2019) have reported that UV irradiation significantly elevates the level of ROS, however, nuclear factor erythroid 2-related factor 2 (Nrf2) overexpression in the skin-derived precursors (SKP) significantly ameliorates UV-induced damage in mice via the remarkable elevation of SOD, CAT, GPx, and reduced glutathione mRNA and protein levels, through Nrf2/HO-1 and PI3K/Akt pathways in a three-dimensional skin model [32]. In addition, it has been reported that the anti-photoaging effects of natural antioxidants, such as sinapic acid [33] and juglanin [34], are mediated by decrease of ROS generation and increases of SOD1, SOD2, CAT, GPx, and Nrf2 gene expression levels in UVB irradiation-damaged HaCaT keratinocytes. Based on the results of the previous studies and our current study, it is likely that LA pretreatment can prevent or attenuate skin damage, by decreasing oxidative stress and increasing antioxidant enzyme levels in mouse dorsal skin. In this regard, further research is required to determine the precise antioxidative mechanisms of LA in photoprotection in the skin.

Antioxidant activities of LA have been demonstrated in several physiological and pathological conditions in which ROS are induced. In rat muscles, LA treatment decreases lipid peroxidation in blood and increases antioxidant levels in the muscles after rigorous exercise in rats [35]. In gerbil hippocampus, after transient cerebral ischemia, LA treatment attenuated superoxide anion generation and lipid peroxidation by increasing the expression of SOD1 and SOD2 [30]. In a rat model of sepsis, LA treatment reduces lipid peroxidation in the lung by maintaining CAT and increasing SOD, GPx and CAT concentration [36]. In addition, low-molecular weight LA treated with gamma irradiation shows superior antioxidant activities, such as 1,1-diphenyl-2-picryl-hydrazyl (DPPH) radical scavenging activity, ferric reducing antioxidant power, and lipid peroxidation inhibition [37]. It has been reported that polysaccharides with lower molecular weight eliminate free radicals effectively, due to the presence of reductive hydroxyl group terminals [38]. Xing et al. (2005) reported that low-molecular-weight chitosan (9 kDa) shows a stronger superoxide anion scavenging effect than high molecular weight chitosan (760 kDa) [39]. Therefore, the studies indicate that LA reduces superoxide anion synthesis by increasing the level of endogenous antioxidant enzymes in the dorsal skin exposed to UVB irradiation.

In conclusion, we demonstrated that the topical application of LA significantly attenuated epithermal thickness and the destruction of dermal collagen fibers in mouse dorsal skin subjected to UVB irradiation, showing that LA treatment significantly reduced ROS production and increased the expression of endogenous antioxidant enzymes in the skin exposed to UVB irradiation. These results suggest that LA prevents UVB-induced skin damage by reducing ROS production and increasing endogenous antioxidant levels against UVB irradiation. In addition, the present results suggest that LA is a useful material for the development of sunscreen products (i.e., cream and/or gel) to protect skin from the damaging effects of UVB exposure. 

## 4. Materials and Methods

### 4.1. Experimental Animals 

Male ICR mice (8-week-old; body weight, 30–40 g) were purchased from Central Lab. Animal Inc. (Seoul, Korea). The animals were housed under conventional housing conditions (temperature, 23 ± 3 °C; relative humidity, 55 ± 5%) under a 12-h light/dark cycle, and they were allowed free access to food and water. The protocol of this experiment, including animal care and handling, was written in compliance with the current international laws and policies (NIH Guide for the Care and Use of Laboratory Animals, NIH Publication, [40]) and approved (approval no. KW-200121-2) by the Institutional Animal Care and Use Committee of Kangwon National University. The numbers of the mice used in this study were minimized, and their suffering by the procedures used in this study was minimized. 

### 4.2. Experimental Groups and LA Treatment

Mice (total *n* = 42) were randomly assigned to three groups (*n* = 14 in each group): (1) control group, which received no LA and UVB exposure; (2) UVB group, which received vehicle (distilled water, DW) and UVB exposure; (3) LA/UVB group, which received LA and UVB exposure.

Hairs of the dorsal skin were shaved four days before UVB irradiation. In the UVB and LA/UVB groups, 200 μL of DW or 3% LA solution was applied to the dorsal skin twice a day for a total of 8 days; simultaneously, UVB exposure was done for 5 days, from the 4th day after LA treatment. The LA used in this study was purchased from Sigma-Aldrich (#L9634, St. Louis, MO, USA) and dissolved in DW at a concentration of 3%. Previous studies demonstrated that long-term (5 weeks) treatment with 1~5% fucoidan, a polysaccharide derived from brown seaweeds, showed the maintenance of the integrity of human cheek skin, without a difference in effect depending on the dosage [41,42]. In this regard, we used 3% LA for 8 days. This method could reduce the number of experimental animals.

In this study, the energy level of UVB intensity was set up as 150 mJ/cm^2^ and 3 min of UVB exposure, which was daily applied with a UVM-225D Mineralight UV Display Lamp (UVP, Phoenix, AZ, USA) for 5 days [15]. The mice in all groups were sacrificed at 5 days after UVB irradiation.

### 4.3. Western Blotting 

Twenty-one mice (*n* = 7 in each group) were used for a Western blot analysis to examine endogenous antioxidants protein levels in mouse dorsal skin. In short, according to a previously published method [43], all mice were deeply anesthetized by an intraperitoneal injection of 60 mg/kg pentobarbital sodium (JW Pharm, Seoul, Korea), and their dorsal skin tissues were collected. These tissues were lysed with RIPA buffer (Santa Cruz, CA, USA) and homogenized with ultrasonic homogenizer for 5 min. These homogenates were centrifuged at 12,000 rpm for 20 min at 4 °C, and the supernatants were collected. Protein concentrations were measured with a bicinchoninic acid kit (Thermo Fisher Scientific, Waltham, MA, USA). The proteins were separated by a solution of 10% sodium dodecyl sulfate–polyacrylamide gel, and transferred to nitrocellulose membranes (Pall Corp., Pittsburgh, PA, USA). These membranes were blocked with 5% non-fat milk solution (in Tris-buffered saline/Tween (TBST)) on a shaker for 60 min at room temperature, and incubated with primary antibodies—rabbit anti-SOD1 (16 kDa) (1:1000, Millipore, Billerica, MA, USA), rabbit anti-SOD2 (24 kDa) (1:1000, Millipore, Billerica, MA, USA), rabbit anti- GPx (22 kDa) (1:1000, Abcam, Cambridge, MA, USA), and rabbit anti-CAT (60 kDa) (1:000, Abcam, Cambridge, MA, USA), and rabbit anti-β-actin (42 kDa) (1:2,000, Sigma-Aldrich, St. Louis, MO, USA) overnight at 4 °C. Additionally, they were washed 3 times with TBST and incubated with peroxidase conjugated anti-rabbit IgG (1:4000, Santa Cruz, CA, USA), for 1 h at room temperature. After washing them with TBST, they were visualized with horseradish peroxidase (Millipore, Billerica, MA, USA). The band intensity was analyzed as relative density using ImageJ (ver. 1.52v, National Institutes of Health, Bethesda, MD, USA).

### 4.4. Preparation of Skin Tissue Sections

Seven mice in each group were deeply anesthetized by intraperitoneal injection of pentobarbital sodium (60 mg/kg; JW Pharm. Co., Ltd, Republic of Korea), at 5 days after UVB irradiation. The whole bodies of the anesthetized mice were transcardially rinsed with 0.1 M phosphate-buffered saline (PBS, pH 7.4) and fixed with a solution of 4% paraformaldehyde (in 0.1 M PB, pH 7.4). The dorsal skin tissues of the mice were removed and post-fixed in the same fixative for one day at room temperature. According to the general method, the fixed skin tissues were dehydrated and embedded in paraffin. Finally, the embedded tissues were sectioned into 8 µm thickness. 

### 4.5. Masson’s Trichrome Staining

To examine changes in the epithermal thickness and density of collagen fibers in the dermis in the dorsal skin, the tissue sections were stained with Masson’s trichrome staining kit (Abcam, Cambridge, MA, USA), as described by Kim et al. [44] The stained sections were prepared as permeant slides. Five sections per each animal were randomly selected to analyze epithermal thickness and the density of collagen fibers. Digital images of the stained tissues were captured using a light microscope (BX53) (Olympus, Tokyo, Japan) and analyzed using Image J (1.46 software, National Institutes of Health, Bethesda, MD).

### 4.6. DHE Staining for Superoxide Anion

To detect an intracellular superoxide anion, the tissues sections were stained with DHE (Sigma-Aldrich, St. Louis, MO, USA), which is a well-known fluorogenic probe. In short, according to previously published studies [30,45], the sections were equilibrated in Krebs-HEPES buffer (composed of 130 mM NaCl, 5.6 mM KCl, 2 mM CaCl_2_, 0.24 mM MgCl_2_, 8.3 mM HEPES, 11 mM glucose, pH 7.4, etc.), for 40 min at 37 °C. Fresh buffer containing DHE (10 μmol/L) was applied on the sections for 2 h at 37 °C, and DHE was oxidized on reaction with superoxide to fluorescent molecule ethidium bromide, which binds DNA in nuclei.

Oxidative stress was analyzed based on the relative fluorescence intensity of DHE, briefly, as described previously by [30]. In brief, digital images of the stained sections were captured with a fluorescence microscope (BX53) (Olympus, Tokyo, Japan), equipped with the excitation wavelength of 520–540 nm. DHE fluorescence intensity was analyzed as RI using Image-pro Plus 6.0 software (Media Cybernetics, MD, USA). The RI was calibrated as %, with the control group (100%).

### 4.7. Immunohistochemistry (IHC)

IHC was conducted to examine endogenous antioxidant immunoreactivity in the dorsal skin, according to our published method [15]. In brief, the tissue sections were immersed in 5% normal goat serum (Vector Laboratories, Inc., Burlingame, CA, USA) (in 0.05 M PBS, pH 7.4), for 30 min at room temperature. Next, these sections were reacted with each diluted primary antibody: rabbit anti-SOD1 (diluted 1:1000, Millipore, Billerica, MA, USA), rabbit anti-SOD2 (1:1000, Abcam, Cambridge, MA, USA), rabbit anti-GPx (1:500, Calbiochem, San Diego, CA, USA), and rabbit anti-CAT (1:500, Calbiochem, San Diego, CA, USA) overnight at 4 °C. Thereafter, these sections were incubated with biotinylated goat anti-rabbit IgG (1:250, Vector, CA, USA) as a secondary antibody. Next, these sections were reacted with avidin-biotin complex (1:300, Vector, CA, USA). Finally, these sections were visualized by responding to them with a solution of 3, 3’-diaminobenzidine tetrahydrochloride (Sigma-Aldrich, St. Louis, MO, USA) (in 0.1 M PBS, pH 7.4). 

To establish the specificity of each immunostaining, each negative control test was done with pre-immune serum instead of each primary antibody. The test showed no immunoreactivity in the observed sections (data not shown).

The immunoreactivity of SOD1, SOD2, GPx and CAT was quantitatively analyzed according to our published method [15]. In short, the digital image of each immunoreactive structure in the dorsal skin was photographed with a light microscope (BX53) (Olympus, Tokyo, Japan). Each image was evaluated as relative optical density (ROD), as follows. Firstly, optical density (OD) was obtained after the transformation of the mean gray level of each immunoreactive image using a formula: OD = log (256/mean gray level). Next, the background was taken from the areas adjacent to the measured areas. Finally, on the basis of the OD of the image, ROD of SOD1, SOD2, GPx and CAT in the dorsal skin were calibrated as %, compared with the control group (100%), using Adobe Photoshop (version 8.0, San Jose, CA, USA) and Image J (1.46 software, National Institutes of Health, Bethesda, MD, USA).

### 4.8. Statistical Analysis 

The data shown in this study represent the means ± standard error of the mean (SEM). Differences of the means among the groups were statistically analyzed by a one-way analysis of variance (ANOVA), with a post hoc Bonferroni’s multiple comparison test carried out to determine differences among groups. Statistical significance was considered at *p* < 0.05.
